# Louisville Summer School of Medicine

**Published:** 1845-09

**Authors:** 


### LOUISVILLE SUMMER SCHOOL OF MEDICINE.

The lectures are resumed in this institution to-day. Professor Gross will deliver regular clinical lectures at the Marine Hospital during the term, and clinical instruction will also be given at the Dispensary under the direction of the Faculty of the Summer School. With the coming of cool weather the dissecting rooms will be furnished, and the most desirable opportunities afforded to students for the prosecution of practical anatomy.

